# Integrative bioinformatics analysis to identify the effects of circadian rhythm on Crohn’s disease

**DOI:** 10.3389/fmolb.2022.961481

**Published:** 2022-09-07

**Authors:** Dan Liu, Yin-Yun Chen, Qing-qing Li, Ming Xu, Jiang-Tao Liao, Ben Wang

**Affiliations:** ^1^ Department of Gastroenterology Medicine, Hunan Provincial People’s Hospital/The First Affiliated Hospital of Hunan Normal University, Changsha, China; ^2^ Department of Dermatology, Xiangya Hospital, Central South University, Changsha, China; ^3^ Hunan Key Laboratary of Aging Biology, Xiangya Hospital, Central South University, Changsha, China; ^4^ National Clinical Research Center for Geriatric Disorders, Xiangya Hospital, Central South University, Changsha, China

**Keywords:** circadian rhythm, Crohn’s disease, bioinformatics integrative analysis, USP2, CR-related gene

## Abstract

**Background:** Crohn’s disease (CD) is a multifactorial inflammatory bowel disease characterized by complex aberrant autoimmune disorders. Currently, the involvement of the circadian rhythm in the pathogenesis of CD is unknown.

**Methods:** Bulk and single-cell RNA-seq data and associated clinical data from patients with CD were downloaded from the Gene Expression Omnibus (GEO). Single-sample gene set enrichment analysis was performed to calculate the enrichment score (ES) of circadian rhythm-related genes. Differential expression analysis was used to identify differentially expressed genes. Functional enrichment analysis was used to explore potential disease mechanisms. CIBERSORT was used to estimate immune cell abundance. Single-cell RNA-seq data were analyzed using the R package “Seurat.”

**Results:** The ES of circadian rhythm-related genes was lower in the CD tissue than in the normal tissue. Ubiquitin-specific protease 2 (*USP2*), a circadian rhythm-related gene, was identified as a potential modulator of CD pathogenesis. *USP2* expression was reduced in CD and was associated with disease severity. Moreover, the analysis of bulk RNA-seq and single-cell RNA-seq data showed that monocyte and neutrophil abundance was elevated in CD and was negatively correlated with *USP2* expression. It should be noted that *USP2* expression in acinar cells was negatively correlated with monocyte and neutrophil abundance. Functional enrichment analysis revealed several canonical pathways to be enriched in CD, including the interleukin-17 signaling pathway, tumor necrosis factor signaling pathway, cytokine–cytokine receptor interaction, toll-like receptor signaling pathway, and nod-like receptor signaling pathway.

**Conclusion:** Aberrant expression of circadian rhythm-related genes is correlated with CD pathogenesis. *USP2* might be related to crosstalk among the different cell types in CD. These findings provide insights into future chronotherapy for CD.

## Introduction

Crohn’s disease (CD) is a multifactorial chronic inflammation of the intestinal segments, that is characterized by ulceration and transmural inflammation (Baumgart and Sandborn, 2012; Molodecky et al., 2012) caused by complex dysregulated interactions between the microbiome and gut immune system. The incidence and prevalence of CD are increasing worldwide and represent a major health burden (Kaplan and Ng, 2017; Ng et al., 2017). However, the clinical benefits of current CD treatments are limited to a subset of patients (Lee et al., 2018). Hence, it is necessary to investigate the molecular mechanism underlying CD pathogenesis and potential biomarkers associated with CD to develop new CD treatment strategies.

Numerous factors, including genetic predisposition, defects in innate immunity, undefined environmental factors, and microbiome alterations, have been proposed to affect the etiology of CD (Kaser et al., 2010; Scarpa and Stylianou, 2012; Strober, 2013; Haberman et al., 2014). One major cause of CD is inadequate activation of the intestinal immune system resulting from a dysfunctional immune response to the enteric microbiota (Scaldaferri and Fiocchi, 2007; Gajendran et al., 2018). Although aberrant immune responses impair intestinal mucosa function and cause damage to the bowel, the specific mechanism underlying autoimmune damage is largely unknown.

Circadian rhythms are 24-h oscillations that control various biological processes in the living system, including two hallmarks of cancer: cell division and metabolism (Papagiannakopoulos et al., 2016). Circadian rhythm-related leukocyte trafficking is dysregulated, thereby contributing to increased systemic low-grade chronic inflammation (Nathan et al., 2021). Some researchers have suggested that irregular shift working confers a risk of contracting inflammatory bowel disease (Sonnenberg, 1990). Circadian disruption, but not sleep loss or stress, may be associated with jet lag-related innate immune system dysregulation and autoimmune diseases (Baxter and Ray, 2020; Olejniczak et al., 2022). Moreover, circadian rhythms are possibly associated with inflammatory bowel disease, but there are no systematic studies or data to support this hypothesis (Gombert et al., 2019). The relationship between CD and the molecular mechanisms underlying its function is not completely understood.

To determine the relationship between circadian rhythms and CD and identify potential mediators of CD pathogenesis, we comprehensively analyzed bulk and single-cell RNA-seq data from patients with CD (GSE93624 (Marigorta et al., 2017), GSE57945 (Loberman-Nachum et al., 2019), and GSE134809 (Martin et al., 2019) datasets. Our findings reveal the association of circadian rhythms with CD and provide an insight into important biomarkers involved in CD development, which will be beneficial for introducing circadian rhythm-oriented treatment strategies for CD.

## Materials and methods

### Public datasets

Bulk and single-cell RNA-seq data and clinical data for patients with Crohn’s disease (CD) were downloaded from Gene Expression Omnibus (GEO), including GSE93624, GSE57945, and GSE134809. The GSE93624 dataset contained 210 treatment-naïve patients of pediatric Crohn’s disease and 35 non-inflammatory bowel disease controls from the risk study and was based on the platform of GPL11154 (Illumina HiSeq 2000). The GSE57945 dataset contained 215 patients with CD and 42 control subjects and was also based on the platform of GPL11154 (Illumina HiSeq 2000). The GSE134809 dataset contained single-cell RNA-seq data from 16 samples (eight CD tissues vs. eight corresponding unaffected tissues) and was based on the platform of GPL18573 (Illumina NextSeq 500). Detailed information refers to the corresponding GSE datasets of the GEO database. Genes in circadian rhythms-related pathways were downloaded from the MSigDB database.

### Differential expression analysis

Differentially expressed genes were calculated using the R package “limma”. The R package “limma” provides a procedure to normalize the data, filter the very low-expression genes, and then implements a series of statistical methods, including empirical Bayes estimation, exact tests, generalized linear models, and quasi-likelihood tests, to calculate the differentially expressed genes. Selection criteria for differentially expressed genes were as follows: |logFC| > 1 and *p* < 0.05.

### Estimation of infiltrating immune cells

Tumor-infiltrating immune cells (TIICs) were estimated using the website tool CIBERSORT (Newman et al., 2015). CIBERSORT is an *in silico* algorithm that enables precise estimation of immune cell fractions using RNA-seq profiles for bulk samples (Li et al., 2020). The accuracy of CIBERSORT has been demonstrated using immunohistochemistry and flow cytometry.

### Single-sample gene set enrichment analysis

Enrichment score (ES) of circadian rhythms for CD and normal tissues was calculated using single-sample gene set enrichment analysis (ssGSEA) based on the R package “GSVA” (Hänzelmann et al., 2013). ssGSEA is a non-parametric and unsupervised method to calculate the variation of gene set enrichment through the samples from an expression dataset. Each ssGSEA enrichment score represents the degree to which the genes in a particular gene set are coordinately up or downregulated within a sample. The key parameters were as follows: kcdf = “Gaussian,” min. sz = 1, max. sz = Inf, tau = 0.25, and abs. ranking = TRUE.

### Functional annotation

Functional annotation was conducted using the R package “clusterProfiler” (version: 3.18.1) (Yu et al., 2012), which provides a comprehensive set of functional annotation tools for researchers to comprehend the biological meaning behind specific gene sets. The clusterProfiler package depends on the Bioconductor annotation data GO. db and KEGG. db to obtain the maps of the entire gene oncology (GO) analysis and Kyoto Encyclopedia of Genes and Genomes (KEGG) corpus. Bioconductor annotation packages org.Hs.eg.db (human genes annotation), org. Mm.eg.db (mice genes annotation), and org. Sc.sgd.db (yeast gene annotation) were imported for genome-wide annotation of mapping Entrez Gene Identifiers or ORF identifiers for humans, mice, and yeast, respectively. Functional annotation consists of the gene ontology (GO) analysis and Kyoto Encyclopedia of Genes and Genomes (KEGG) pathways, which allows investigating what biological functions and signaling pathways are involved. We also conducted gene set enrichment analysis (GSEA) based on a ranked gene set using “clusterProfiler”. The GSEA analysis could reveal some enriched signaling pathways missed in the GO and KEGG analyses. The key parameters were as follows: pAdjustMethod = “BH,” pvalueCutoff = 0.05, q-valueCutoff = 0.2, nPerm = 1,000, minGSSize = 10, and maxGSSize = 500.

### Statistical analysis

All statistical analyses were completed using R software (Version 4.0.1). Single-cell RNA-seq data were analyzed using the R package Seurat, a toolkit for quality control, analysis, and exploration of single-cell RNA sequencing data. “Seurat” aims to enable users to identify and interpret sources of heterogeneity from single-cell transcriptomic measurements and integrate diverse types of single-cell data. Based on the data homogeneity of variance and normal distribution, either the independent sample *t*-test or Wilcoxon signed-rank test was chosen. Student’s *t*-test is used for data conformity to normal distribution and homogeneity of variance; otherwise the Wilcoxon signed-rank test is used. Spearman’s correlation coefficient was used to assess the correlation between two continuous variables. *p* < 0.05 was considered statistically significant.

## Results

### Relationship between circadian rhythm and Crohn’s disease

**FIGURE 1:**
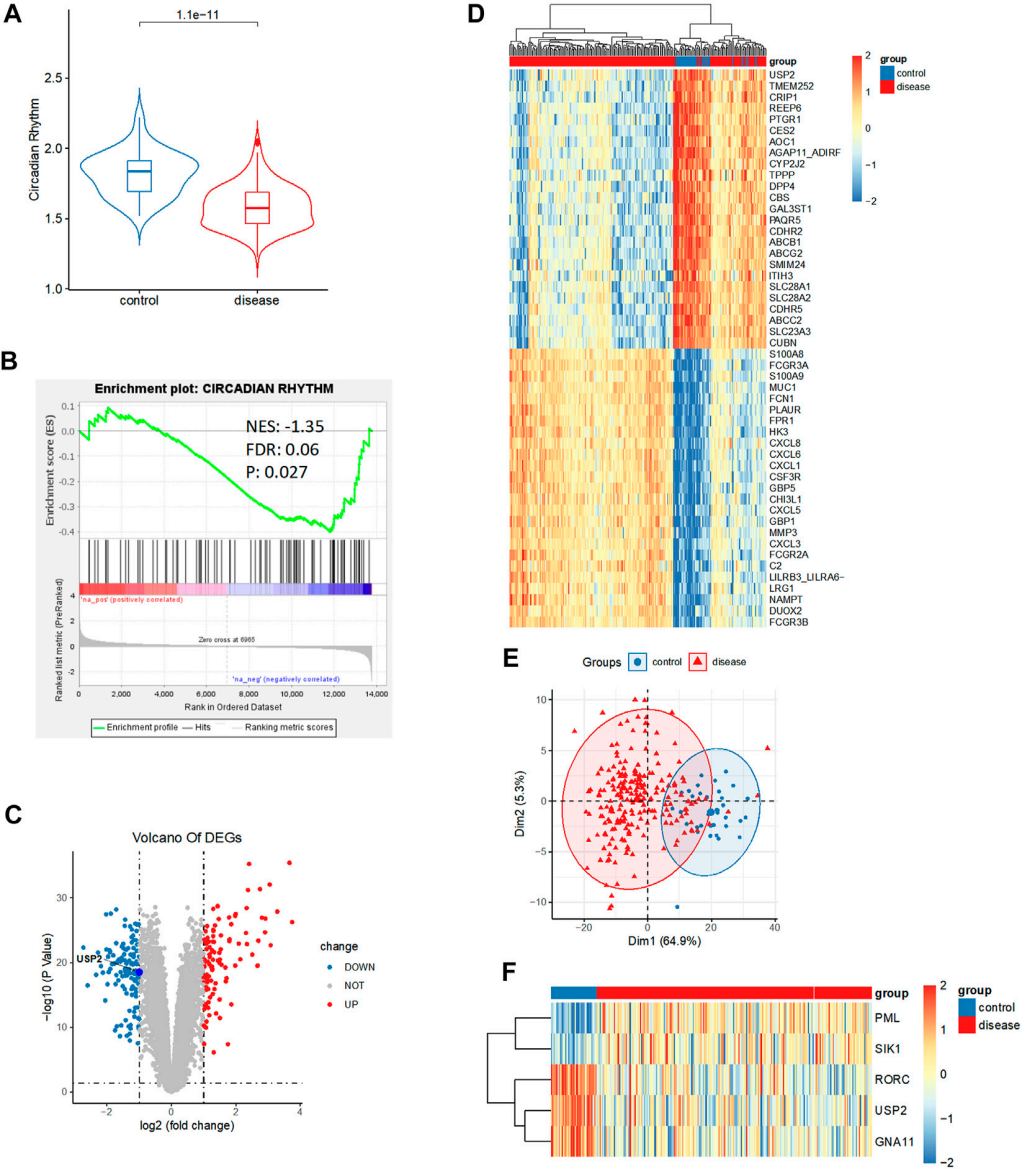
Relationship between circadian rhythm and Crohn’s disease (CD). **(A)** Enrichment score of circadian rhythm-related genes was significantly elevated in control than in CD tissues. **(B)** Circadian rhythm was significantly downregulated in CD. **(C)** Volcano plot of 229 differentially expressed genes between control and CD tissues, including 109 upregulated genes and 120 downregulated genes (|logFC| > 1, *p* < 0.05). **(D)** Heatmap analysis showed a distinct expression pattern between the control and CD groups. **(E)** Principal component analysis repeatedly displayed a distinct expression pattern between the control and CD groups. **(F)** Heatmap analysis showed circadian rhythm expression patterns between the control and CD groups.

Circadian rhythm is associated with many biological processes, including cell division and metabolism (Papagiannakopoulos et al., 2016). Therefore, we investigated the association of circadian rhythm with CD. To identify the relationship between circadian rhythm and CD, we compared the enrichment score (ES) of circadian rhythms between CD tissues and control intestinal tissues from the GSE93624 dataset. The ESs of circadian rhythm-related genes were significantly higher in the control tissues than in the CD tissues (*p* < 0.05; Figure 1A). To validate this relationship, we performed gene set enrichment analysis (GSEA) of circadian rhythm-related genes using bulk RNA-seq data from the CD samples. Consistently, the expression of circadian rhythm-related genes was significantly downregulated in CD samples (normalized enrichment score [NES] = −1.35, false discovery rate [FDR] = 0.06, and *p* = 0.027; Figure 1B), suggesting a potential role for circadian rhythm in CD.

To identify the important genes involved in CD development, we conducted a differential expression analysis between the control and CD groups in the GSE93624 dataset. We identified 229 differentially expressed genes, consisting of 109 upregulated genes and 120 downregulated genes (|logFC| > 1, *p* < 0.05; Figure 1C and Supplementary Table S1). We performed heatmap analysis and principal component analysis (PCA) to test for the presence of a distinct expression pattern between the control and CD groups. We observed a distinct expression pattern between the control and CD groups (Figures 1D,E). Among the 117 circadian rhythm-related genes (Supplementary Table S2), the expression of 81 genes was statistically different, but the fold difference was very weak. Only five genes had a relatively large fold difference (|logFC| > 0.5). The heatmap of the expression of these five genes showed that three genes displayed a significantly downregulated expression and two genes displayed a significantly upregulated expression in CD (Figure 1F and Supplementary Table S3). It should be noted that we observed that ubiquitin-specific protease 2 (*USP2*), a circadian rhythm-related gene with the largest significant difference, was present in the downregulated genes.

### Functional enrichment analysis of differentially expressed genes

**FIGURE 2:**
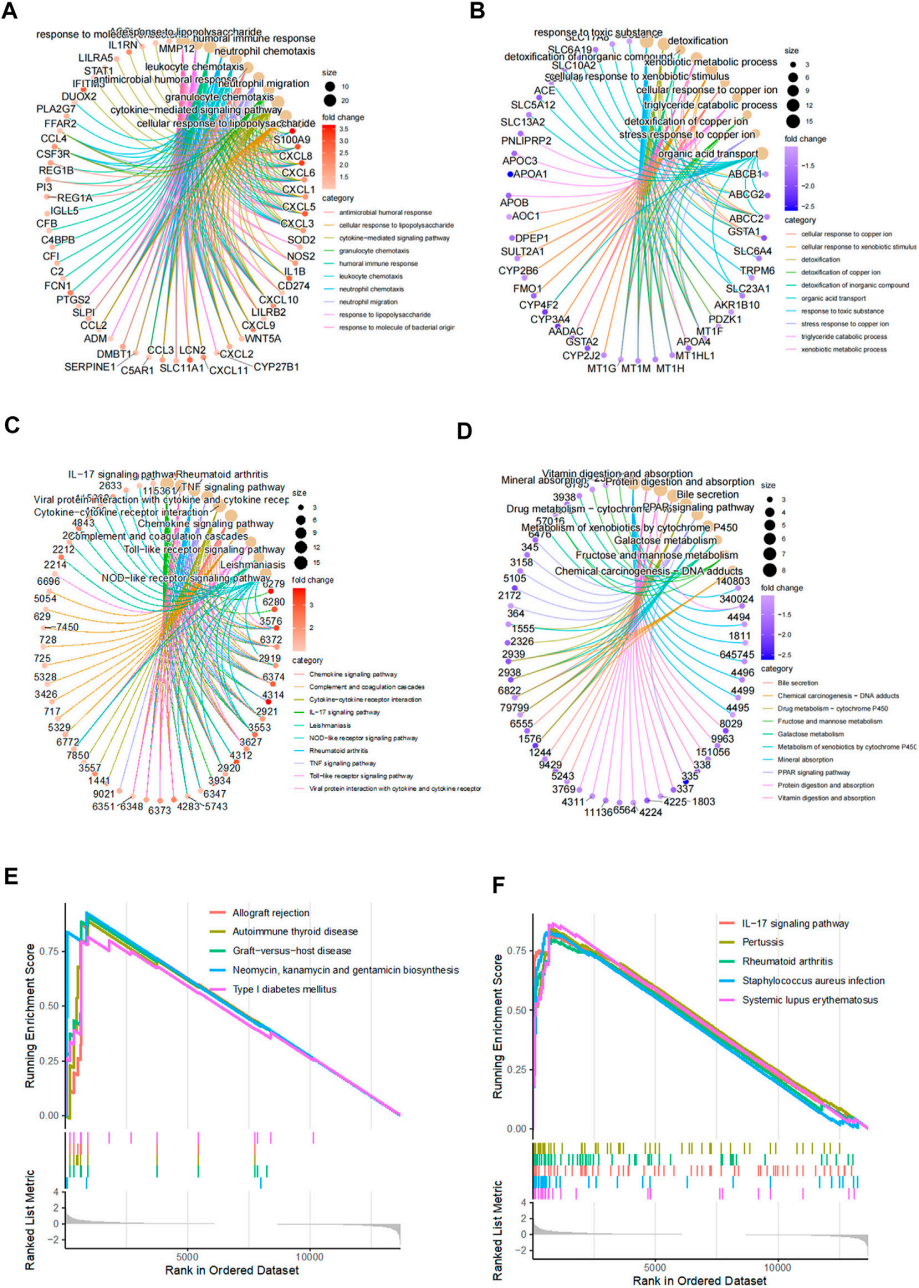
Functional enrichment analysis for differentially expressed genes. **(A–B)** Top 10 upregulated and downregulated GO terms in CD disease. **(C–D)** Top 10 upregulated and downregulated KEGG pathways in CD disease. **(E–F)** Top 10 upregulated KEGG pathways generated by gene set enrichment analysis.

Considering the fact that the previous results showed a distinct gene expression pattern in CD, we investigated the molecular mechanism underlying CD development by performing gene ontology (GO) and the Kyoto Encyclopedia of Genes and Genomes (KEGG) pathway enrichment analysis for 109 significantly upregulated genes and 120 significantly downregulated genes between the control and CD groups. The top 10 enriched GO terms included response to molecules of bacterial origin, response to lipopolysaccharide, humoral immune response, neutrophil chemotaxis, and neutrophil migration, suggesting a hyperactivated immune state (Figures 2A,B). Consistently, the top 10 enriched KEGG pathways included interleukin-17 signaling pathway, tumor necrosis factor signaling pathway, cytokine–cytokine receptor interaction, toll-like receptor signaling pathway, and nod-like receptor signaling pathway (Figures 2C,D). Gene set enrichment analysis also identified enriched immune-related signaling pathways, including allograft rejection, graft-versus-host disease, and interleukin-17 signaling pathway (Figures 2E,F), suggesting that CD is an autoimmune disorder.

### Investigation of the immune microenvironment in CD

**FIGURE 3:**
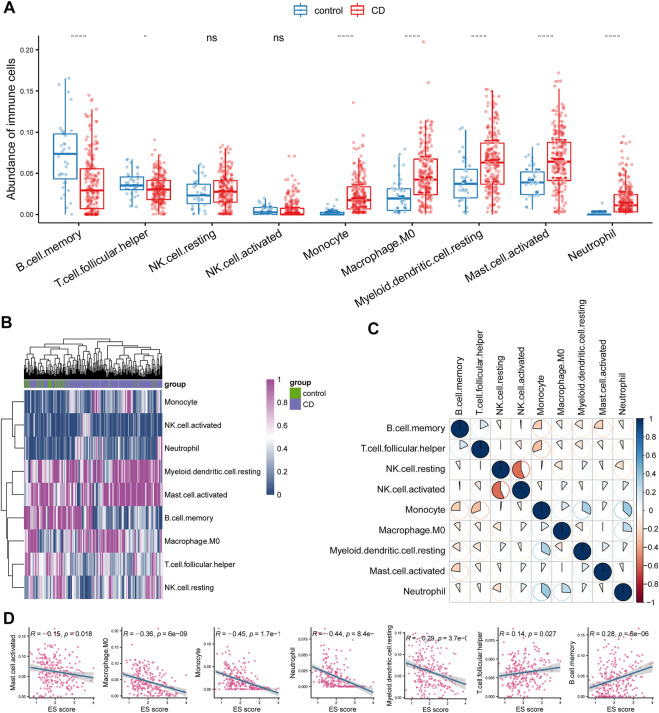
Investigation of immune microenvironment of CD. **(A)** Comparison of the abundance of immune cells between control and CD tissues. **(B)** Heatmap analysis also indicated a distinct expression of these cells between the groups. **(C)** Correlation analysis between different immune cell types in CD. **(D)** Enrichment score (ES) of circadian rhythm was negatively correlated with monocyte, M0 macrophage, myeloid dendritic cell, activated mast cell, and neutrophil whereas positively correlated with B cell and T cell follicular helper.

Considering that the functional enrichment analysis indicated the involvement of hyperactivated immunity in CD, we analyzed changes in immune cell abundance between control and CD tissues using bulk RNA-seq data. Immune cell abundance was estimated using the CIBERSORT algorithm. We observed that the counts of monocytes, M0 macrophages, myeloid dendritic cells, activated mast cells, and neutrophils were significantly upregulated, whereas those of B cells and T follicular helper cells were significantly downregulated in CD (Figure 3A). Furthermore, neutrophil counts were elevated in CD, which was in concordance with the functional enrichment analysis results. Heatmap analysis also indicated that the counts of these cells were different between the sample groups (Figure 3B). Correlation analysis showed that the presence of neutrophils was significantly positively correlated with that of monocytes (Figure 3C). Consistently, we found that ESs of circadian rhythm-related genes were negatively correlated with the abundance of monocytes, M0 macrophages, myeloid dendritic cells, activated mast cells, and neutrophils. These scores were positively correlated with the abundance of B cells and T follicular helper cells (Figure 3D; *p* < 0.05).

### Association of *USP2* with CD

**FIGURE 4:**
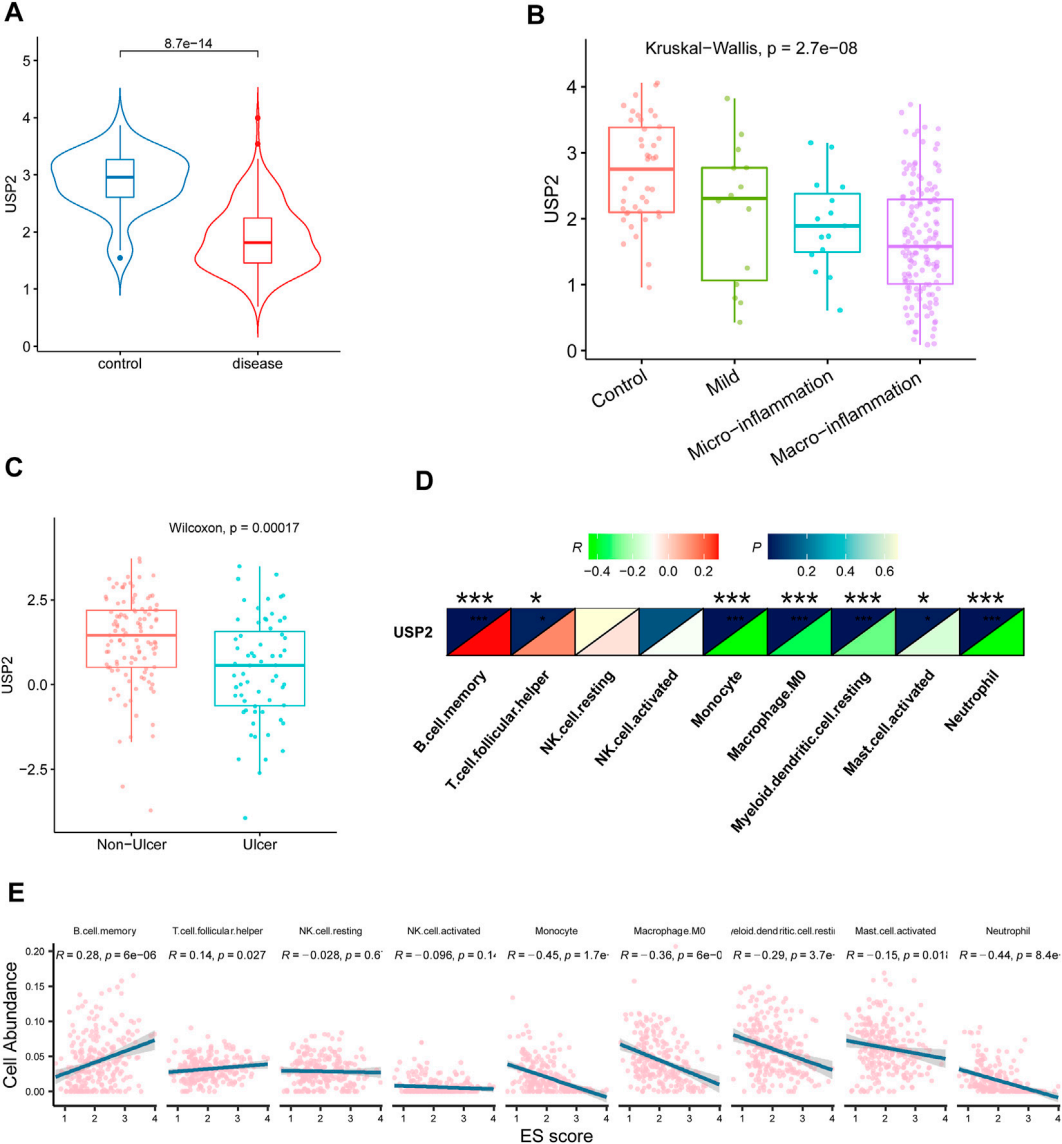
Association of *USP2* with CD. **(A)** *USP2* was also downregulated in CD compared with the control group in an independent cohort of CD patients from GSE57945. **(B)** *USP2* was associated with the severity of disease; the expression of USP2 decreased as the disease progressed. **(C)** *USP2* was downregulated in CD with ulcers than without ulcers. **(D–E)** *USP2* was negatively correlated with monocyte, M0 macrophage, myeloid dendritic cell, activated mast cell, and neutrophil whereas positively correlated with B cell and T cell follicular helper.

Our results thus far indicate a potential association between *USP2* and CD. We sought to validate the relationship between *USP2* expression and CD using bulk RNA-seq data from an independent cohort (GSE57945). *USP2* expression was downregulated in the CD tissues (*p* = 8.7e-14; Figure 4A). Moreover, *USP2* expression was also found to be associated with disease severity, and *USP2* expression decreased as the disease progressed (Figure 4B). Accordingly, *USP2* expression was downregulated in the tissues of patients with CD with ulcers compared to that in tissues of patients with CD without ulcers (Figure 4C). Consistent with immune cell-related changes in CD, *USP2* expression was negatively correlated with the counts of monocytes, M0 macrophages, myeloid dendritic cells, activated mast cells, and neutrophils and positively correlated with the counts of B cells and T follicular helper cells (Figures 4D,E; *p* < 0.05).

### Validating the dysregulation of *USP2* expression in single immune cells

**FIGURE 5:**
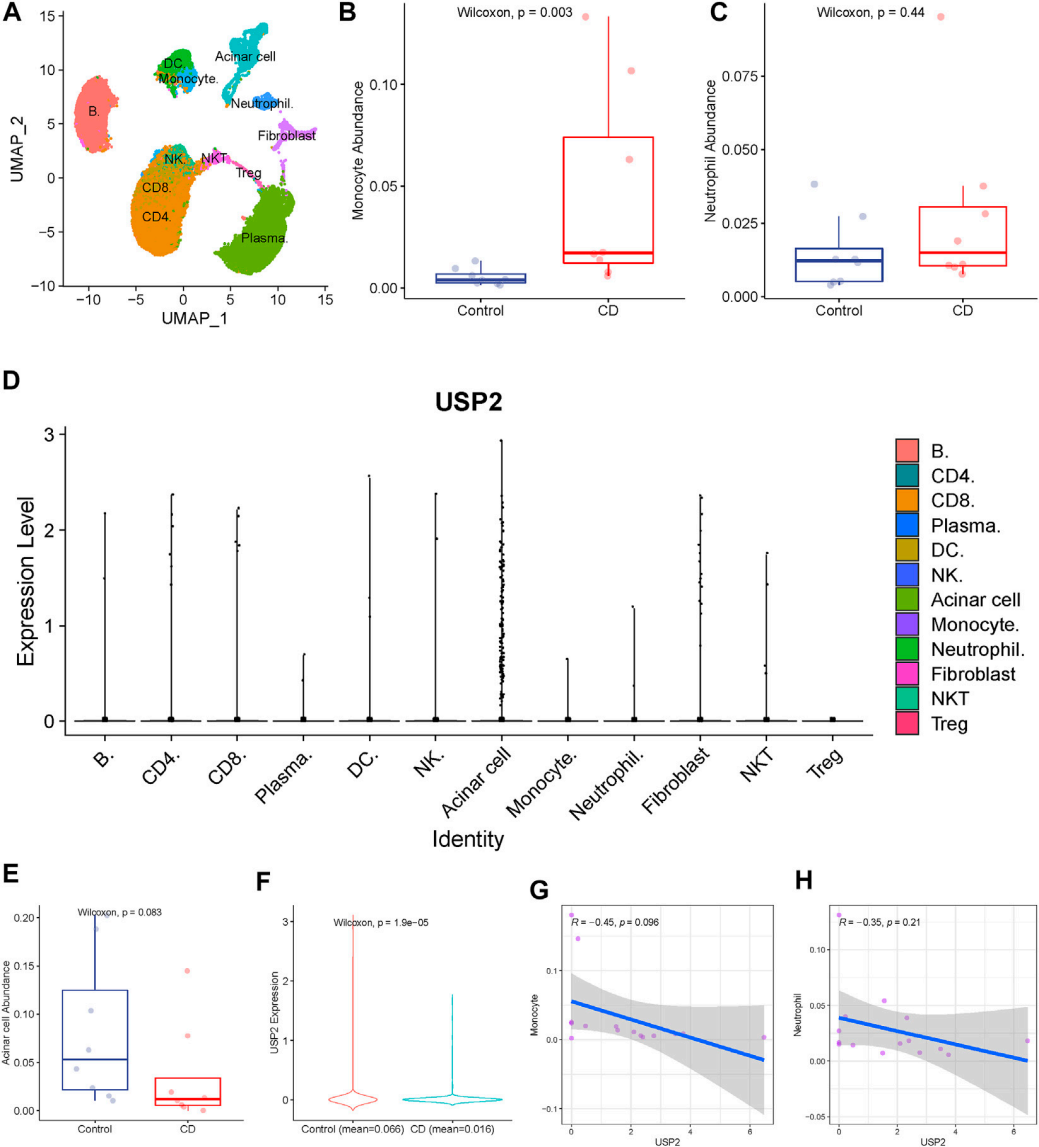
Validation of the association of *USP2* with immune cells. **(A)** Total of 60,228 cells from 16 samples were clustered into 12 subsets. **(B)** Monocyte was upregulated in CD than in the control group. **(C)** *USP2* expression levels were compared across different cell types, and *USP2* has the highest expression value in acinar cells. **(D)** Acinar cell was upregulated in control than in CD, although there was no significant difference (Wilcoxon test; *p* = 0.083). **(E)** *USP2* was also upregulated in acinar cells of the control group than in the CD group. **(G–H)** *USP2* expression in acinar cells was negatively correlated with the abundance of monocyte and neutrophil, although there was no significant difference.

To validate the relationship between *USP2* and immune cells at the single-cell level, we analyzed the association between *USP2* expression and immune cells using single-cell RNA-seq data from eight CD tissues and their paired normal ileal tissues (from the GSE134809 dataset). A total of 60,228 cells from 16 samples were clustered into 12 subsets composed of acinar cells, monocytes, neutrophils, and B cells (Figure 5A). Among them, two cell types (monocytes and neutrophils) were identical to those identified using CIBERSORT. Therefore, we compared their abundance between the control and CD groups. Consistent with the results from the GSE93624 cohort, single-cell RNA-seq data showed that counts of monocytes and neutrophils were increased in the CD group compared to those in the control group (Figures 5B,C), although neutrophil counts displayed no significant difference between the CD and control groups.

We then compared *USP2* expression levels across different cell types. *USP2* had the highest expression in acinar cells (Figure 5D), indicating its role in normal intestinal tissue. Therefore, we compared acinar cell abundance between the CD and control groups and found that acinar cell counts were downregulated in the CD group compared to those in the control group, although there was a borderline significant difference (Wilcoxon test; *p* = 0.083; Figure 5E). *USP2* expression was also significantly downregulated in acinar cells of the CD group compared to that in the acinar cells of the control group (Figure 5F), further highlighting its possible role in acinar cells.

Next, we investigated the crosstalk between different cell types. In particular, to determine the link between *USP2* expression and the abundance of monocytes and neutrophils in CD, we analyzed the relationship between *USP2* expression in acinar cells and monocyte and neutrophil abundance. Interestingly, *USP2* expression in acinar cells was negatively correlated with monocyte and neutrophil abundance, although this difference was only slightly significant (Figures 5G,H), suggesting that *USP2* expression may be related to crosstalk among acinar cells, monocytes, and neutrophils in CD.

## Discussion

In this study, we identified the association between circadian rhythm and CD. We also identified a circadian rhythm-related gene, *USP2*, as a potential mediator of CD pathogenesis. Moreover, we observed that monocyte and neutrophil counts were significantly elevated in CD and were negatively associated with circadian rhythm and *USP2* expression, suggesting their role in CD pathogenesis. These findings provide a new rationale for development of a circadian rhythm-oriented treatment for CD.

Disturbances in circadian rhythm are involved in several inflammatory diseases (Bechtold et al., 2010; Logan and Sarkar, 2012; Bollinger and Schibler, 2014; Maury et al., 2014). Here, we hypothesized and validated the role of circadian rhythm-related genes in CD and its association with immune function. We found that the ES of circadian rhythm-related genes was significantly higher in control tissues than in CD tissues. ES represents the expression of a particular gene set in different individuals. The decreased ES of circadian rhythm-related genes suggests that circadian rhythms are closely related to CD. Circadian rhythm is involved in the regulation of immune functions. Functional molecular clocks have been described in splenic macrophages, dendritic cells, natural killer cells, and B cells (Arjona et al., 2004; Silver et al., 2012; Yu et al., 2013). Approximately 8% of macrophages exert their function in a circadian fashion, which could have profound regulatory effects on immune function (Maury et al., 2014). Moreover, cytokine release is partly shifted in response to changes in the sleep–wake cycle, which might contribute to increased risk of autoimmune diseases in shift workers (Cuesta et al., 2016). The present study also revealed the involvement of monocytes and neutrophils in CD, underlining their potential roles in the etiology of CD. These findings further support the idea that disruption of circadian rhythm is involved in exacerbated immune responses in the bowel.

Another substantial finding of this study is the potential of *USP2* as an important biomarker for CD. *USP2* is a circadian rhythm-related gene that is involved in multiple biological processes, such as pressure overload-induced cardiac remodeling (Xing et al., 2020), breast cancer (He et al., 2019), hemophilia (Xu et al., 2021a), and energy metabolism (Kitamura and Hashimoto, 2021). *USP2* is a multifunctional deubiquitinating enzyme that controls cell cycle progression and carcinogenesis *via* cyclin and Aurora-A deubiquitination (Lin et al., 2020; VanGenderen et al., 2020). Moreover, *USP2* serves as an important component of the CLOCK/BMAL1 complex and mediates rhythmic gene expression in the suprachiasmatic nucleus and the liver (Xu et al., 2021b). Furthermore, *USP2* promotes surface expression of ion channels in renal and intestinal epithelial cells (Zhou et al., 2013; Rougier et al., 2015), which is consistent with our findings that *USP2* is associated with acinar cells in CD patients. In addition to modifying cytokine production in immune cells, *USP2* modulates the signaling molecules involved in cytokine signaling in target cells (Totzke et al., 2020; Xing et al., 2020). Accordingly, our functional enrichment analysis results showed that the cytokine–cytokine signaling pathway was enriched in CD.

This study has a few limitations that warrant further research. The key genes and signaling pathways in this study were identified using bioinformatics analysis. Therefore, further *in vitro* and *in vivo* studies are required to explore the associated physiological mechanisms of action. Additionally, gene rhythmicity (i.e., variations in gene expression depending on the time of tissue sampling) should be considered in future studies.

In conclusion, this study describes the disturbed circadian rhythm-related gene expression patterns in CD, reveals the association between circadian rhythm and CD, and provides potential biomarkers involved in CD development. These biomarkers will be beneficial for developing a circadian rhythm-oriented treatment strategy for CD. This study adds new evidence to support the relationship between circadian rhythm and intestinal diseases and proposes the potential role of *USP2* in intestinal diseases, thereby providing a target for subsequent molecular interventions.

## Data Availability

The datasets presented in this study can be found in online repositories. The names of the repository/repositories and accession number(s) can be found below: https://www.ncbi.nlm.nih.gov/, GSE93624; https://www.ncbi.nlm.nih.gov/, GSE57945; and https://www.ncbi.nlm.nih.gov/, GSE134809.
